# 2025 European Society of Cardiology guidelines for the management of cardiovascular disease and pregnancy: Statement of endorsement by the NVVC

**DOI:** 10.1007/s12471-026-02085-z

**Published:** 2026-10-15

**Authors:** Heleen B. van der Zwaan, Deborah N. Kalkman, Marjolijn J. Broekman, Annemiek C. C. Evers, Bernardine H. Stegeman, Philippine Kiès, Caroline M. van der Marel, Judith van der Vloed, Iris C. D. Westendorp, Jérôme M. J. Cornette

**Affiliations:** 1 Department of Cardiology, University Medical Center Utrecht, Utrecht, The Netherlands; 2 Department of Cardiology, Amsterdam University Medical Center, Amsterdam, The Netherlands; 3 Department of Obstetrics and Gynaecology, University Medical Center Utrecht, Utrecht, The Netherlands; 4 Knowledge Institute of the Federation of Medical Specialists, Utrecht, The Netherlands; 5 Department of Cardiology, Heart Lung Center, Leiden University Medical Center Leiden, Leiden, The Netherlands; 6 Department of Anesthesia, Erasmus MC, University Medical Center Rotterdam, Rotterdam, The Netherlands; 7 Dutch Heart Foundation, Den Haag, The Netherlands; 8 Department of Cardiology, Red Cross Hospital, Beverwijk, The Netherlands; 9 Department of Obstetrics and Fetal Medicine, Erasmus MC, University Medical Center Rotterdam, Rotterdam, The Netherlands

**Keywords:** Pregnancy, Cardiovascular disease, Pregnancy heart team, Congenital heart disease, Aortopathies, Peripartum

## Abstract

This article contextualises the 2025 European Society of Cardiology (ESC) guidelines for managing cardiovascular disease (CVD) and pregnancy in the Netherlands. The guideline addendum provides additional recommendations for situations where the ESC guidelines may not fully align with Dutch clinical practice. By endorsing the ESC guidelines through this addendum, the Netherlands Society of Cardiology (Nederlandse Vereniging Voor Cardiologie) supports its members in adhering to evidence-based management strategies for CVD and pregnancy.

## Introduction

The 2025 European Society of Cardiology (ESC) Guidelines for the management of cardiovascular disease (CVD) and pregnancy provide an overview of all available information regarding pregnancy and CVD [1]. Since the previous version of these guidelines was published in 2018, [2] new scientific evidence has been gathered, and additional clinical insights have been obtained, resulting in a revised version of the guideline. This revision includes the growing role of the Pregnancy Heart Team, advice regarding the use of contraception, methods for pre-pregnancy counselling and risk stratification to support shared decision-making with the patient, and the use of diagnostic tests and medications during pregnancy and lactation.

In this statement of endorsement, we summarize the most important new findings and explain the adaptations we have made for Dutch clinical practice (Tab. 1).

**Table 1:** Modifications to the 2025 European Society of Cardiology guidelines for the management of cardiovascular disease and pregnancy by the writing group.

ESC guideline	Class	LOE	Table	Update for Dutch clinical practice—recommendation*
*Chapter 4 The Pregnancy Heart Team*				
Discontinuation of lactation for 24 h should be considered in women in whom i.v. gadolinium is required	IIa	C	2	Due to the limited excretion of contrast media (CM) in breast milk, continuation of breastfeeding after administration of CM is deemed safe, in line with Dutch guidelines. If the patient herself wishes to interrupt breastfeeding (shared decision-making with the physician), then a period of 24 h is sufficient. (Class III; Level C)
Routine induction of labour prior to 39 weeks is not recommended in women with stable CVD	IIa	C		In women with CVD, planned induction around term (39–40 weeks) should be considered to optimize multidisciplinary care and reduce the risk of unplanned delivery. It is offered to all women after informed consent. In women preferring expectant management after counseling, patient preference is taken into account, and an individualized plan is established
–	I	C		A peripartum care plan should contain the following: 1. Medication safety during pregnancy/lactation. 2. Timing, mode & location of delivery. 3. Anesthesia strategies. 4. Anticoagulation. 5. Delivery & postpartum plan
*Chapter 5 Drugs during pregnancy and lactation*				
DOACs are not recommended during pregnancy	III	C	4	This recommendation has been withdrawn. Instead, please refer to the Dutch Guideline Antitrombotisch beleid
Chapter 6 *Pregnancy in women with cardiomyopathies and primary arrythmia syndromes*				
Sotalol may be considered as an anti-arrhythmic drug in pregnant women with ARVC, with careful evaluation of QTc and while monitoring for foetal bradycardia and foetal growth and neonate hypoglycaemia	IIb	C	5	Sotalol (second line option after flecainide) may be considered as an anti-arrhythmic drug in pregnant women with ARVC, with careful evaluation of QTc and while monitoring for foetal bradycardia and foetal growth and neonate hypoglycaemia. Given the substantial transfer of Sotalol to the breast milk and the neonate, restraint or caution is advised for breast feeding during Sotalol therapy
	IIa	C		
–	IIa	C	6	Anesthesiologic involvement in the Pregnancy Heart team is desirable in the second trimester. The possibilities and appropriateness of epidural analgesia should be discussed. Effective epidural analgesia in high-risk cardiac patients can help reduce the maternal stress response and hemodynamic consequences during labor
*Chapter 7 Peripartum cardiomyopathy*				
Adding at least prophylactic LMWH treatment to bromocriptine treatment in women with PPCM should be considered	IIa	C	7	Adding at least prophylactic LMWH treatment to bromocriptine or cabergoline treatment in women with PPCM should be considered
Bromocriptine may be considered in addition to optimal HF treatment to enhance recovery of LV function in women with PPCM	IIb	B		Bromocriptine or cabergoline treatment may be considered in addition to optimal HF treatment to enhance recovery of LV function in women with PPCM
*Chapter 8 Pregnancy in women with aortapathies*				
Beta-blocker therapy throughout pregnancy and in the post-partum period is recommended in women with MFS and other HTADs	I	C	8	This recommendation has been withdrawn. Instead, please refer to the Dutch Guideline Marfan syndroom
In women with MFS and aortic root diameters > 45 mm, surgery before pregnancy is recommended	I	C	8	This recommendation has been withdrawn. Instead, please refer to the Dutch Guideline Marfan syndroom
In women with MFS and aortic root diameters between 40 and 45 mm, surgery before pregnancy may be considered if risk factors (growth > 3 mm/year, family history of aortic dissection) are present	IIb	C	8	This recommendation has been withdrawn. Instead, please refer to the Dutch Guideline Marfan syndroom
In women with vascular Ehlers-Danlos syndrome, caesarean section at 37 weeks is recommended for obstetrical reasons	I	C	8	In women with vascular Ehlers-Danlos syndrome, caesarean section at 37 weeks is recommended for cardiac reasons and for obstetrical reasons. In women with vascular Ehlers-Danlos syndrome who express a preference for vaginal delivery, an individualized management approach may be considered after multidisciplinary counselling [3]
Cardiac surgery may be considered during pregnancy when conservative and medical therapy has failed, and in situations that threaten the mother’s life or that are not amenable to percutaneous treatment	IIb	C	8	This recommendation has been withdrawn, as this is considered self-evident
*Chapter 10 Pregnancy in women with pulmonary arterial hypertension*				
For women with PAH requiring pregnancy termination, it is recommended to perform this in PH centres	I	C	10	For women with PAH requiring pregnancy termination, it is recommended to perform this in PH centres or in centres connected to these PH centres
*Chapter 11 Venous thromboembolism in pregnancy and post-partum*				
For pregnant or post-partum women at high risk^c^ of VTE, a prophylactic fixed dose of LMWH is recommended over a higher weight-adjusted dose to reduce the risk of VTE	I	B	11	This recommendation has been withdrawn. Instead, please refer to the Dutch Guideline Antitrombotisch beleid
In pregnant women or women in the post-partum period with suspicion of VTE (DVT and/or PE), an immediate formal diagnostic assessment with validated methods is recommended and should not be postponed	I	B	11	In pregnant women or women in the post-partum period with suspicion of VTE (DVT and/or PE), an immediate formal diagnostic assessment with validated methods (following the Dutch Guideline, see module Diagnostics VTE) is recommended and should not be postponed
In pregnant or post-partum women with a diagnosis of VTE without haemodynamic instability, anticoagulation is recommended by using therapeutic-dose LMWH based on early pregnancy body weight	I	C	11	This recommendation has been withdrawn. Instead, please refer to the Dutch Guideline Antitrombotisch beleid
In pregnant women or women in the post-partum period with a strong clinical suspicion of VTE, initiation of treatment with a therapeutic dose of LMWH should be considered until the presence of VTE has been ruled out or confirmed	IIa	C	11	In pregnant women or women in the post-partum period with a strong clinical suspicion of VTE, initiation of treatment with a therapeutic dose of LMWH should be considered until the presence of VTE has been ruled out or confirmed, provided the bleeding risk is acceptable
In pregnant or post-partum women with a diagnosis of acute high-risk PE, a catheter-based reperfusion strategy or systemic thrombolysis should be considered	IIa	C	11	In pregnant or postpartum women with a diagnosis of acute high-risk PE, a reperfusion strategy (systemic thrombolysis, catheter-based reperfusion or surgical thrombectomy) is recommended (choice is dependent on the local situation and expertise)
In pregnant or post-partum women with a diagnosis of acute high-risk PE, surgical thrombectomy may be considered as an alternative to a catheter-based approach or systemic thrombolysis	IIb	C	11	This recommendation has been integrated into the previous recommendation
*Chapter 12 Pregnancy in women with acquired heart disease*				
Continuation of statins may be considered during pregnancy in women with established ASCVD	IIb	C	12	Continuation of statins may be considered during pregnancy in women with established ASCVD or with a very high risk of CVD, such as homozygous familial hypercholesterolemia
In severe hypertension, drug treatment with i.v. labetalol, urapidil, nicardipine, or oral short acting nifedipine or methyldopa is recommended for acute reduction in blood pressure. Intravenous hydralazine is a second-line option	I	C	13	In severe hypertension, drug treatment with i.v. labetalol, urapidil, nicardipine, or oral short acting nifedipine is recommended for acute reduction in blood pressure
In women with gestational hypertension, delivery is recommended at 39 weeks	I	B	13	In women with gestational hypertension, delivery is recommended between 37 and 39 weeks
Immediate caesarean section at the site of the arrest should be considered and immediately prepared if ROSC has not been achieved in the mother after 4 min of resuscitative efforts and if the foetus is viable, taking gestational age, comorbidities, and the available level of medical care into account	IIa	C	16	Immediate caesarean section at the site of the arrest should be considered and immediately prepared and performed if the foetus is viable, above 20 weeks of gestation, taking comorbidities, and the available level of medical care into account
–	I	C	18	To emphasize the importance of the pregnancy heart team, the following recommendation was added: Ensure that, during pre-pregnancy counseling of patients with native valve disease, timely consultation with the valvular heart team takes place regarding possible valve intervention
It is recommended that the type of valve surgery or intervention for a woman contemplating pregnancy is chosen in consultation with the Pregnancy Team	I	C	19	It is recommended that the type of valve surgery or intervention for a woman contemplating pregnancy is chosen with the valvular heart team considering a possible future pregnancy
–	I	C	23	It is recommended to send a medical discharge letter to the general practitioner for all women with adverse pregnancy outcome (APO), to guide cardiovascular risk management

## Summary and translation into clinical practice in the Netherlands

### Physiology of pregnancy

**Fig. 1:**
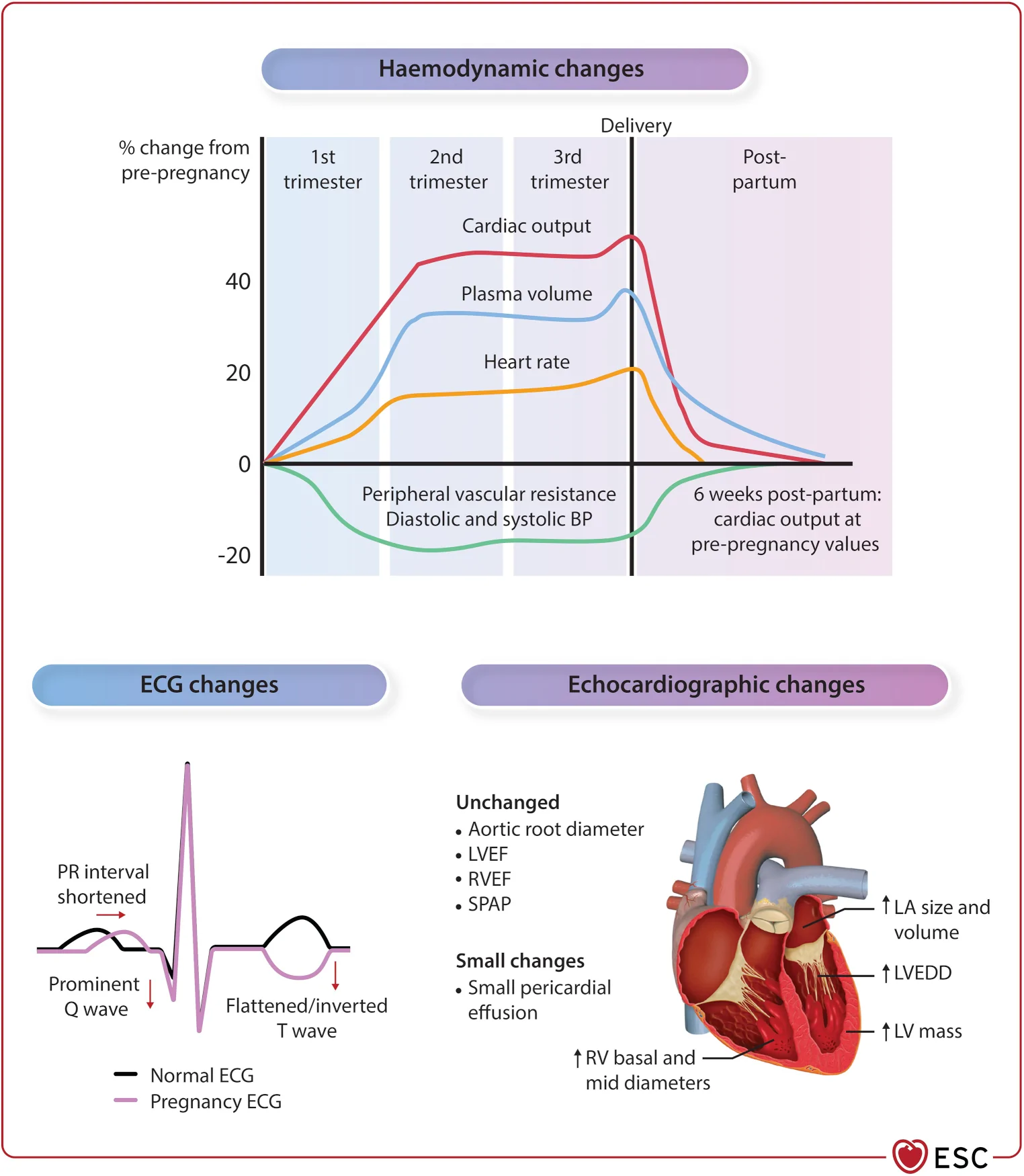
Physiology of haemodynamic changes, and changes in electrocardiogram and echocardiography during and post pregnancy, *from the ESC guidelines* [1]

From the first trimester of pregnancy onward, hemodynamic changes occur, including increases in cardiac output, plasma volume, and heart rate (Fig. 1; [4]). Blood pressure decreases due to a reduction in peripheral vascular resistance. These changes generally stabilize during the second trimester and become most pronounced around the time of delivery [5]. In the absence of heart disease, physiological changes on the electrocardiogram may occur, such as a more prominent Q wave and T‑wave inversion. Furthermore, as a result of pregnancy and associated hemodynamic shifts, the left atrium may dilate, the left and right ventricles may enlarge, and left ventricular (LV) mass can increase [6].

### Pre-pregnancy counselling and evaluation

#### mWHO 2.0 Risk assessment

Pre-pregnancy counselling aims to assess the extent to which the maternal heart can adapt to the altered hemodynamic state and the increased cardiac workload it imposes. To achieve this, the modified World Health Organization (mWHO) 2.0 risk classification can be used. It encompasses a wider spectrum of CVD during pregnancy than the previous version and distinguishes between different categories: ventricular dysfunction and pulmonary hypertension, arrhythmias, cardiomyopathies, congenital heart disease, valvular heart disease, aortopathies, and acquired/coronary and other CVD (Tab. 2). It provides an estimated maternal cardiac event rate and subsequently advises on Pregnancy Heart Team involvement and the location of cardio-obstetric care during pregnancy and delivery. The Cardiac Disease in Pregnancy study II (CARPREG II) score can subsequently be used as a risk modifier.

**Table 2:** Modified World Health Organization 2.0 classification of maternal cardiovascular risk, *from the ESC guidelines* [1].

		mWHO 2.0 I	mWHO 2.0 II	mWHO 2.0 II–III	mWHO 2.0 III	mWHO 2.0 IV
*Diagnosis*		*Ventricular (dys)function+pulmonary hypertension*				
				Mild left ventricular impairment: EF > 45%. Significantly impaired RV (subpulmonary) function	Moderate left ventricular impairment: EF 30%–45%. Previous PPCM with not more than mild residual left ventricular impairment	Severe left ventricular impairment: EF < 30% or NYHA class III/IV. Previous PPCM with more than mild left ventricular impairment. PAH
		*Arrhythmias*				
		Atrial or ventricular ectopic beats, isolated	Most supraventricular arrhythmias. Bradycardia requiring pacemaker	Low-risk LQTS: no previous events + on full dose beta-blocker therapy. Low-risk CPVT: well controlled by medical therapy. BrS with no previous events	Sustained ventricular tachycardia from any aetiology. LQT2 (post-partum). Symptomatic CPVT and LQTS not adequately controlled by therapy. BrS with previous events	
		*Cardiomyopathy*				
		HCM: genotype-positive + phenotype-negative		Low-risk ARVC: genotype-positive + no or mild phenotype. HCM without complications. DCM/NDLVC with normal or mild left ventricular impairment: EF > 45%	ARVC with moderate/severe disease. HCM with arrhythmic and/or moderate haemodynamic complications. DCM/NDLVC with moderate left ventricular impairment: EF 30%–45%	DCM/NDLVC with severe left ventricular impairment: EF < 30% or NYHA class III/IV. HCM with symptomatic severe outflow tract obstruction: ≥ 50 mm Hg. HCM with severely symptomatic LV dysfunction (EF < 50%)
		*Congenital heart disease*				
		Successfully repaired simple lesions without significant residual (haemodynamic) complications (atrial or ventricular septal defect, patent ductus arteriosus, anomalous pulmonary venous drainage)	Unoperated uncomplicated atrial or ventricular septal defect. Repaired tetralogy of Fallot without significant residual haemodynamic/arrhythmic lesions. Transposition of the great arteries with arterial switch without significant residual lesions	Repaired atrioventricular septal defect without significant residual lesions. Uncomplicated Ebstein anomaly: mild to moderate TR, no tricuspid stenosis, no accessory pathway	Unrepaired cyanotic heart disease (not Eisenmenger). Systemic RV with good or mildly decreased ventricular function. Uncomplicated Fontan circulation: good ventricular function, no significant valve disease or arrhythmias, good exercise tolerance, and normal arterial saturations. Ebstein anomaly with any complication	Systemic RV with moderate or severely decreased ventricular function. Fontan with any complication. Eisenmenger syndrome
		*Valvular heart disease*				
		Small or mild – pulmonary stenosis – mitral valve prolapse without significant regurgitation		Native, homograft or tissue valve disease not considered mWHO 2.0 I or IV: mild mitral stenosis, moderate aortic stenosis. Moderate valvular regurgitation	Uncomplicated mechanical valve with stable well controlled INRs. Moderate mitral stenosis. Severe asymptomatic aortic stenosis. Severe left-sided valvular regurgitation	Severe mitral stenosis. Severe symptomatic aortic stenosis
*Diagnosis*		*Aortopathy*				
		Non-HTAD mild aortic dilatation (< 40 mm)	Turner syndrome without cardiovascular features (BAV, coarctation, AHT, aortic dilatation)	Marfan or other HTAD syndrome without aortic dilatation. Aorta < 45 mm in BAV pathology. Repaired coarctation	Moderate aortic dilatation: 40–45 mm in Marfan syndrome or other HTAD; 45–50 mm in BAV, Turner syndrome ASI 20–25 mm/m^2^, other aortic dilatation < 50 mm. Marfan with previous aortic root replacement. Previous aortic dissection with stable diameter	Severe aortic dilatation: > 45 mm in Marfan syndrome or other HTAD, > 50 mm in BAV, ASI > 25 mm/m^2^ in Turner syndrome, other aortic dilatation > 50 mm. Vascular Ehlers-Danlos syndrome. Severe (re)coarctation. Previous aortic dissection with increasing diameter
		*Acquired+coronary heart disease+other*				
					Prior SCAD. Prior ischaemic cardiac event (STEMI/NSTE ACS). Prior adverse pregnancy outcome requiring hospitalization. Prior adverse cardiovascular effects of cancer treatment	
*Risk*		No detectable increased risk of maternal mortality and no/mild increased risk in morbidity	Small increased risk of maternal mortality or moderate increase in morbidity	Intermediate increased risk of maternal mortality or moderate to severe increase in morbidity	Significantly increased risk of maternal mortality or severe morbidity	Extremely high risk of maternal mortality or severe morbidity
Average maternal cardiac event ratesa	Van Hagen et al. (2016)	9.9%	7.7%	17.7%	28.9%	50.3%
	Silversides et al. (2018)	3.1%	21.7%	12.8%	21.1%	35.6%
*Individualize each maternal risk with the modifiers below*b *(derived from CARPREG II)* [52]						
*CARPREG II score: 1 point* No prior cardiac intervention indicated Late pregnancy assessment			*CARPREG II score: 2 points* Ventricular dysfunction High-risk left-sided valve disease or outflow tract obstruction Pulmonary hypertension Coronary artery disease High-risk aortopathy		*CARPREG II score: 3 points* Prior cardiac event or arrhythmias Baseline NYHA III/IV or cyanosis Mechanical valve	
*Involvement of the Pregnancy Heart Team*		No	No	Yes	Yes	Yes
Counselling		Yes (by regular healthcare professional)	Yes (by regular healthcare professional)	Yes: expert counselling by Pregnancy Heart Team is required	Yes: expert counselling by Pregnancy Heart Team is required	Yes: expert counselling by Pregnancy Heart Team is required, with clear and thorough discussion of very high pregnancy risk and shared decision-making process for termination if pregnancy occurs
*Obstetric and cardiac care during pregnancy*		Local hospital	Local hospital	Shared care with local hospital + Pregnancy Heart Team	Care led by Pregnancy Heart Team	Care led by Pregnancy Heart Team
*Location of delivery*		Local hospital	Local hospital	Shared care with local hospital + Pregnancy Heart Team. Location depends on CV status and evolution of pregnancy	Expert centre, care led by Pregnancy Heart Team	Expert centre, care led by Pregnancy Heart Team

*AHT* arterial hypertension, *ARVC* arrhythmogenic right ventricular cardiomyopathy, *ASI* aortic size index, *BAV* bicuspid aortic valve, *BrS* Brugada syndrome, *CARPREG II* Cardiac Disease in Pregnancy study II; *CPVT* catecholaminergic polymorphic ventricular tachycardia, *CV* cardiovascular, *DCM* dilated cardiomyopathy, *EF* ejection fraction, *HCM* hypertrophic cardiomyopathy, *HTAD* heritable thoracic aortic diseases, *INR* international normalized ratio, *LQTS* long QT syndrome, *LQT2* long QT syndrome type 2; *LV* left ventricle, *mWHO* modified World Health Organization; *NDLVC* non-dilated left ventricular cardiomyopathy, *NSTE ACS* non-*ST* -elevation acute coronary syndrome, *NYHA* New York Heart Association, *PAH* pulmonary arterial hypertension, *PPCM* peripartum cardiomyopathy, *RV* right ventricle, *SCAD* spontaneous coronary artery dissection, *STEMI ST* -elevation myocardial infarction, *TR* tricuspid regurgitation

^a^Definition of cardiac events: cardiac arrest, cardiac death, arrhythmia requiring treatment, left/right heart failure, thromboembolic event, aortic dissection, acute coronary syndrome, or hospitalization for cardiac reason. Endocarditis only in van Hagen *et al.* [51]

^b^Estimation of maternal adverse cardiac event rate with integration of CARPREG II score. Reprinted from Silversides *et al.* [52] with permission from Elsevier

The recommendation to perform risk assessment using the mWHO 2.0 risk classification in all women with CVD of childbearing age has been fully endorsed by the working group. In Dutch clinical practice, women with an mWHO 2.0 classification above class II should be evaluated and counselled by the Pregnancy Heart Team from pre-pregnancy to pregnancy and delivery.

#### Pre-pregnancy counselling

In adolescents with known CVD, starting from menarche, education on maternal and fetal risk should be provided, preferably during transition programs. In women with a risk of thrombo-embolic events, progestin-only treatment, contraceptive implants, and/or levonorgestrel IUDs should be considered.

During an extensive preconception counselling consultation—preferably conducted by at least a gynaecologist and a cardiologist in patients with more severe heart disease—the physiological adaptations of the heart during pregnancy can be discussed, as well as the maternal risks (for example, the development of heart failure or arrhythmias) and fetal risks (such as preterm birth or low birth weight). Genetic counselling, lifestyle counselling, reproductive technologies, drug review and pregnancy termination should be discussed when relevant. In expertise centres with multidisciplinary teams, pre-implantation and prenatal genetic testing are available in pregnancies in which one or both parents have known monogenic or chromosomal abnormality. In the Netherlands, PGT‑M is nationally coordinated through Maastricht UMC+, in collaboration with clinical genetics centers.

Infertility rates are similar in women with and without CVD [7]. However, all women with CVD planning to use assisted reproduction should have an individual risk assessment for venous thrombo-embolism. Moreover, the Dutch addendum writing committee added a recommendation that single-embryo transfer be performed in women with CVD and that moral reasoning/ethical reflection (in Dutch ‘Moreel beraad’) be undertaken beforehand in complex cases.

In pregnant women with mWHO 2.0 risk classification IV, the option of termination of pregnancy should be considered and discussed based on maternal and/or fetal risks. In these counselling sessions, professional psychological support should be provided.

### Patient perspective

The ESC guidelines emphasize respect for the woman’s autonomy by incorporating her perspective in decisions regarding contraception, pregnancy, delivery, and lactation. Maternal health literacy and preferences should be thoroughly explored as part of the shared decision-making process. Following publication of the 2025 guidelines, the clinical practice guideline “Management of Cardiovascular Disease and Pregnancy: What Patients Need to Know” was published. This document offers patient information on aspects such as delivery, breastfeeding, and long-term maternal care. The aim of the document is to give patients confidence to collaborate with healthcare professionals in making well-informed decisions.

To achieve the best possible discussion about increased risks and to stimulate shared decision-making, it is important to provide the patient and her relatives with clear information on a regular basis. Addressing contraception options and a (possible future) desire for pregnancy encourages the patient and her relatives to reflect on these issues and to formulate their preferences. Determining whether these preferences align with what is medically feasible and safe is an important topic of discussion. During pregnancy, routine follow-up visits are scheduled, including consultations with the cardiologist and obstetrician, with a frequency depending on the underlying cardiovascular disease and the presence or development of symptoms. Here too, it is important to take the patient’s preferences into account to tailor care as closely as possible to her needs. For these patients, it is important to align care closely with the involved healthcare providers and clear communication about the organisation, coordination and continuity of care.

### Pregnancy Heart Team

The Pregnancy Heart Team provides risk assessment, an individual care plan, monitoring of pregnancy and education for patient. A primary midwifery-led care system and secondary and tertiary medical specialist-led care systems characterize the Dutch obstetric care system. The Dutch hospital system consists of separate secondary and tertiary medical care centres with a well-organized and structured referral system between them. Pregnancy and delivery are characterized by substantial hemodynamic challenges, as well as unpredictable course and timing. Thus, intrapartum care is often provided by a multidisciplinary on-call team whose composition may vary.

In the 2025 ESC guidelines, the Pregnancy Heart Team has received an more prominent role in optimizing outcomes for women with CVD during pregnancy. In the Dutch context, CVD is considered a medical indication for medically supervised care during pregnancy and delivery, underscoring the need for structured interdisciplinary collaboration. The guideline recommends that all women with CVD of mWHO 2.0 class II–III and above be evaluated and managed by a dedicated Pregnancy Heart Team from pre-pregnancy through postpartum follow-up.

Based on recent Dutch guidance outlining how to establish and operationalize Pregnancy Heart Teams, tertiary care centres serve as hubs of expertise with integrated teams that include cardiologists, obstetricians, anaesthetists as well as geneticists, haematologists, neonatologists, intensivists, cardiovascular surgeons, specialized nurses, and allied professionals as required. Standardized processes, consistent documentation, and 24/7 accessibility of reports in the electronic health record ensure reliable communication and decision-making across professionals involved. After each meeting, the patient’s electronic record is updated, and the cardiologist or maternal-fetal medicine specialist informs the patient and/or referring physician of the team’s conclusions and care plan.

All women with conditions classified as mWHO 2.0 class II–III, III, or IV are recommended to be managed in such dedicated tertiary centres. For selected women with mWHO 2.0 class II–III disease who are clinically stable and in whom risks can be adequately mitigated, coordinated care within a dedicated secondary care centre may be considered, always in close collaboration with the tertiary Pregnancy Heart Team. For women with mWHO 2.0 class I, II or II–III conditions managed in secondary hospitals, we also recommend multidisciplinary discussion within a local Pregnancy Heart Team. If a formal Pregnancy Heart Team is not established in a secondary care facility, an individualized care plan for pregnancy and delivery should be developed and jointly discussed and approved by the local gynaecologist, cardiologist, and anaesthetist, with a low threshold for consultation with the tertiary Pregnancy Heart Team. This ensures the most appropriate care at the most appropriate site and avoids unprepared on-call teams and unnecessary emergency referrals during labour and delivery. In occasional cases with low risk, shared care with primary midwifery care can be considered during pregnancy according to local protocols. As a large part of postpartum home care is provided by primary care stakeholders (midwives and home maternity nurses) good information exchange with all parties is essential.

In the Dutch context, a well-structured Pregnancy Heart Team in close collaboration with local stakeholders contributes to safer pregnancies and more equitable, high-quality care for women with cardiovascular disease at the most optimal site.

### Peripartum care plan

It is important to gather information from the patient in a timely manner to understand her wishes and needs regarding peripartum care. The primary goal is a safe pregnancy for both mother and child; therefore, not all wishes or requests can always be accommodated.

**Fig. 2:**
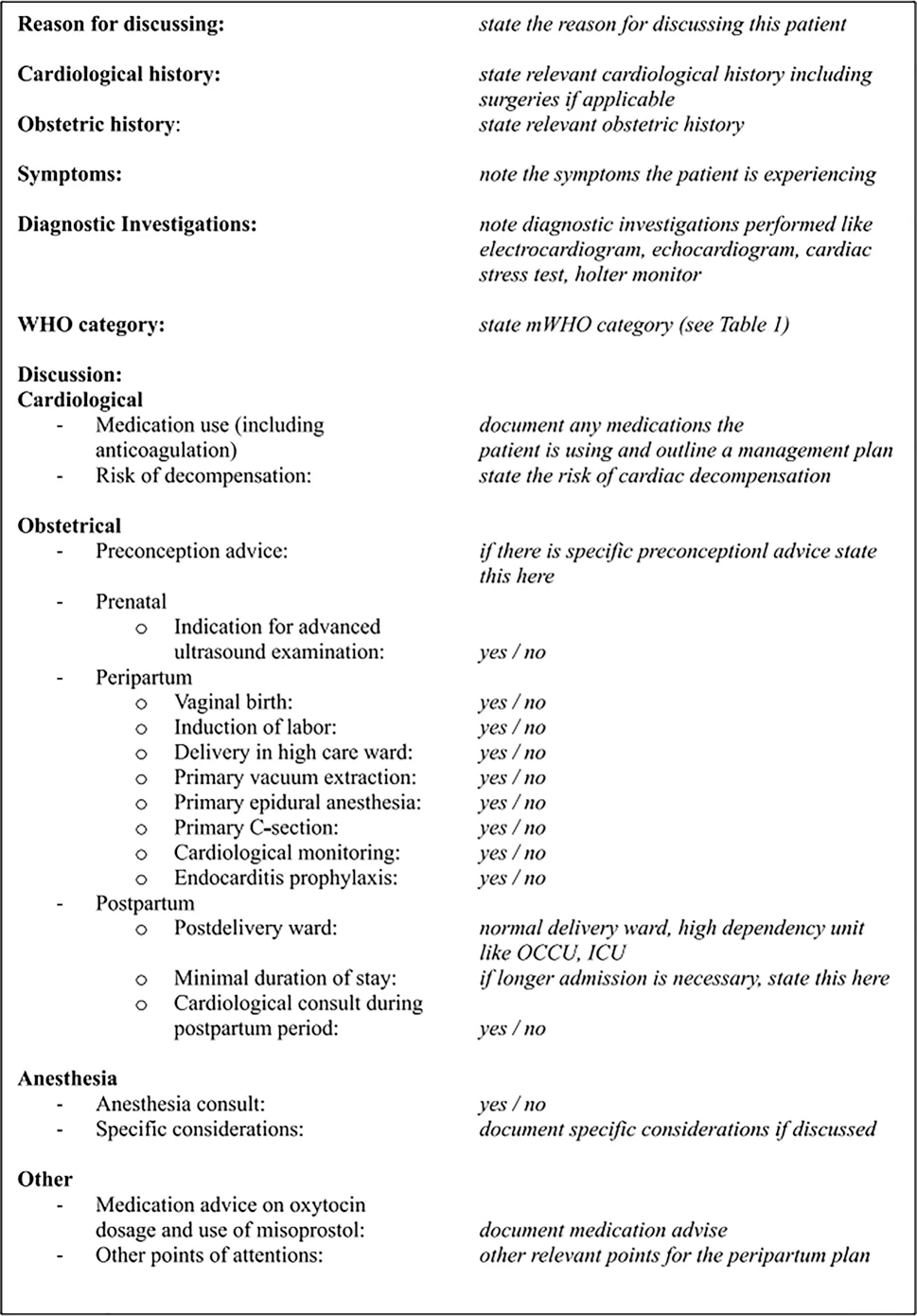
A peripartum care plan including all relevant topics, *from Rijs et al*. [8]

Anaesthesiology involvement during pregnancy is important for women with CVD, and an early consultation, preferably in the second trimester, can help prepare an individualised plan for a safe delivery (Fig. 2; [8]). A central part of this preparation is discussing the options for pain relief and anaesthesia. For women who aim for a vaginal delivery, it is important to consider whether epidural analgesia is desirable and possible. Epidural analgesia can attenuate the maternal cardiac stress response and promote greater hemodynamic stability during labour. This may be particularly beneficial for women with valvular disease, stress-related arrhythmias, or impaired cardiac function, who may poorly tolerate tachycardia or abrupt blood pressure fluctuations. By attenuating these stress responses, epidural analgesia can contribute to a safer labour process. In addition, epidural analgesia can facilitate assisted vaginal delivery when needed. For example, in women for whom active pushing or Valsalva manoeuvres are not advisable, epidural analgesia can facilitate vacuum-assisted delivery. This helps limit maternal exertion and prevents large hemodynamic fluctuations. If a caesarean section becomes necessary, it is advantageous to have discussed the choice between neuraxial and general anaesthesia in advance. For many women with cardiac disease, neuraxial anaesthesia may provide greater hemodynamic stability, although general anaesthesia may be appropriate in selected situations.

In many women with CVD, planned induction around term (39–40 weeks) should be considered to optimize multidisciplinary care and reduce the risk of unplanned delivery [9–12]. All women with CVD deserve nuanced counseling on this matter after which induction is offered. If a woman expresses a preference for expectant management, her preference, choice and autonomy is respected, and an individualized plan is made.

### Drugs during pregnancy and lactation

Given the hemodynamic changes during pregnancy, pharmacokinetics and pharmacodynamics differ during pregnancy (Fig. 1; [5, 13]). Teratogenic effects, the risk of transmission through the placenta, and transmission during lactation are all considerations for pregnant and breastfeeding women. The 2025 guidelines provide recommendations regarding the use of medication during pregnancy and lactation, including the safest choice, alternative options, and medication to avoid.

The use of anticoagulants during pregnancy is highlighted, given the complex balance between risks and benefits. For the Dutch context, the writing group refers to the Dutch antithrombotic guideline with regard to antithrombotic therapy. Patients with mechanical heart valves who require a low dose of vitamin K antagonists (VKA) may be advised to continue VKA therapy during the first trimester. As the risk of embryopathy is dose-dependent, switching to low-molecular-weight heparin (LMWH) in cases of higher VKA doses is recommended for the first trimester. Importantly, therapeutic LMWH should only be used in centers where anti-Xa monitoring is available. Women with mechanical heart valves still carry a high risk of adverse pregnancy outcomes [14, 15].

Given the substantial transfer of sotalol to breast milk and the neonate, restraint or caution is advised with breastfeeding. Women with peripartum cardiomyopathy (PPCM) cabergoline treatment (as an alternative to bromocriptine) may be considered in selected patients in addition to optimal heart failure treatment to enhance recovery of LV function due to a better safety profile, although clinical evidence remains limited.

### Pregnancy in women with specific CVD

#### Peripartum cardiomyopathy

Peripartum cardiomyopathy (PPCM) is a potentially life-threatening condition defined as heart failure with a left ventricular ejection fraction of < 45% without any other cause of heart failure, that occurs mainly during the peripartum period. Recent trials suggest a ‘multiple hit’ theory for developing PPCM, with an accumulation of genetic and environmental risk factors. There is growing evidence that several pathophysiological mechanisms in PPCM converge on a common pathway, which involves inflammation, oxidative stress imbalance, and endothelial dysfunction.

Treatment of acute heart failure caused by PPCM follows the main principles of heart failure management during and after pregnancy. The management strategy should be discussed with the Pregnancy Heart Team, considering maternal, fetal, and neonatal outcomes. In addition to heart failure treatment, bromocriptine or cabergoline, which suppress prolactin production, may be considered in women with moderate to severe heart failure in PPCM. Furthermore, adding LMWH should be considered to reduce the thromboembolic risk. When a reversible course of heart failure is assumed, treatment in accordance with heart failure guidelines should be considered for at least 12 months after complete LV recovery. Genetic testing should be considered in women with PPCM.

Counselling for women with PPCM about risk of recurrence during a subsequent pregnancy and about contraception is recommended in all cases, even after full recovery of LV function (LV ejection fraction > 50%). There is a strong recommendation to continue beta-blocker therapy, and reinitiating beta-blocker therapy before a subsequent pregnancy may be beneficial, irrespective of baseline LV systolic function.

### Aortopathies

In the ROPAC III study, the incidence of aortic dissection was 3.5% in pregnant women with heritable thoracic aortic disease [16]. Fortunately, this incidence is lower than previously expected. However, when there is a desire for pregnancy, different thresholds for prophylactic surgical aortic treatment prior to pregnancy are considered to minimize the risk of aortic dissection during pregnancy. Moreover, in patients with aortopathy, it is important to have recent measurements of the aorta, preferably by magnetic resonance angiography or computed tomography angiography of the aorta, to adequately monitor potential growth during pregnancy and by echocardiogram, if feasible. The current guideline provides an overview of the threshold for prophylactic surgical aortic root and ascending aneurysms prior to pregnancy and the recommended mode of delivery. The guidelines do not specify how recent the aortic measurement should be. In women with vascular Ehlers-Danlos syndrome not only vascular complications may occur, but also lacerations and pre-term delivery. Life threatening complications include arterial dissection or rupture, uturine rupture, and surgical complications. Most complications occur in women who were unaware of their diagnosis at the time of pregnancy [17].

For patients with Marfan syndrome, the working group refers to the Dutch Marfan guidelines.

### Congenital heart disease

The increasing number of adults with congenital heart disease (ACHD) has led to a rise in pregnancies among women with ACHD. The prospective Registry of Pregnancy and Cardiac Disease (ROPAC) gathered data on CVD and pregnancy and revealed improving outcomes and generally favourable maternal and fetal outcomes in women with ACHD [18]. However, maternal, obstetric and fetal risk differ depending on the underlying condition (such as left ventricular outflow tract obstruction, shunts, pulmonary valve and RVOT disease, repaired Tetralogy of Fallot, Ebstein’s anomaly, transposition of the great arteries, single ventricle physiology palliated with Fontan circulation and unrepaired cyanotic ACHD (without pulmonary hypertension)). The guideline provides a structured overview of pregnancy management and delivery.

### Acquired heart disease

In contrast to the previous guidelines, increasing attention is given to acquired heart disease, such as coronary artery disease (CAD), hypertensive disorders, arrhythmias, valvular disease and heart failure.

The target blood pressure values during pregnancy have been tightened: systolic blood pressure should be below 140 mm Hg and diastolic below 90 mm Hg [19]. Recommendations are provided for additional diagnostics, as well as non-pharmacological and pharmacological treatment options. Methyldopa, labetalol, and/or nifedipine are preferred medications. In women with gestational hypertension, delivery is recommended at 37 to 39 weeks of gestation.

New clinical data from ROPAC and PPCM registries led to revised management strategies [7, 20–28]. For instance, continuation of beta-blockers in patients with heart failure, previously recommended during pregnancy (Class I), is now suggested for consideration (Class IIa). Conversely, in patients with hypertrophic cardiomyopathy, beta-blockers are now recommended to be continued (Class I), rather than merely considered (Class IIa). Importantly, patients with cardiomyopathy must be informed that pregnancy can lead to irreversible deterioration of LV function [29].

In women with atherosclerotic CVD during pregnancy, low-dose acetylsalicylic acid is the recommended antiplatelet treatment of choice. When dual antiplatelet therapy (DAPT) is required in pregnant women undergoing coronary stent implantation or experiencing a myocardial infarction, the addition of clopidogrel is the treatment of choice, and the duration of DAPT is recommended to be the same as in non-pregnant women, with an individual approach considering ischemic risk and delivery-related bleeding risks. The continuation of statins may be considered during pregnancy in women at very high risk [30–32]. In the Netherlands, given the low risk of adverse fetal effects, continuation of statins during pregnancy is often practiced and should therefore be carefully considered.

For arrhythmias, including inherited arrhythmia syndromes, the guidelines state clear advice on medication during pregnancy, and especially in complex cases, evaluation in the Pregnancy Heart Team or adding an electrophysiologist to the team is in place. To minimize inappropriate shocks during delivery in ICD patients, the VT zone should be turned off temporarily, with only the VF zone remaining active.

### Acute cardiac care during pregnancy

The incidence of CVD during pregnancy is increasing, due to both inherited and acquired CVD rising in women of reproductive age [33, 34]. As suggested in the section Pregnancy Heart team, pregnant women with known CVD will be evaluated during regular visits and strategies to avoid deterioration of CVD are deployed. However, acute cardiac care could be needed in both women with known and unknown CVD during pregnancy.

One of the key messages of the 2025 ESC guidelines is that *‘In a life-threatening situation, treatments such as defibrillation, interventions, acute coronary revascularization, mechanical circulatory support, and medication should be the same as in non-pregnant women, irrespective of contraindications*.’ [35]. The purpose is to avoid any delay of adequate cardiac care in life-threatening situations.

However, in non-life-threatening but acute situations, an emergency multidisciplinary approach is needed with involvement of a cardiologist, gynaecologist, intensive care doctor and/or other required specialties, depending on the type of CVD or treatment strategy. The best location for the patient during hospital stay (cardiac care unit/intensive care) as well as the best treatment strategy (balancing maternal and fetal interest) should be evaluated, based on the disease and the stage of gestation. The guideline provides an overview of medication considerations, as well as strategies for the treatment of cardiomyopathies and primary arrhythmia syndromes, congenital heart disease, venous thromboembolism and women with acquired heart disease (such as acute coronary syndrome), arrhythmias, valvular disease, heart failure and hypertension [35].

### Adverse pregnancy outcomes and follow-up

For women who experience adverse pregnancy outcomes— such as hypertensive complications, gestational diabetes, preterm birth, or pregnancy loss—it is recommended to extend postpartum care to reduce long-term CVD risks. Close collaboration between the gynaecologist, cardiologist, obstetric physician, and general practitioner is essential to ensure appropriate long-term care.

## Conclusion

This endorsement of the 2025 ESC guidelines on the management of CVD during pregnancy provides an overview of key recommendations for daily clinical practice in the Netherlands, while also highlighting sources for more detailed information on pregnancy and CVD. The addendum is also published on the Guideline database of the Dutch Association of Medical Specialists (www.richtlijnendatabase.nl).

## Data Availability

All data supporting the findings of this work are available from the corresponding author upon reasonable request.

## References

[1] De Backer J, Haugaa KH, Hasselberg NE, et al. 2025 ESC Guidelines for the management of cardiovascular disease and pregnancy. Eur Heart J. 2025;46(43):4462–568. 10.1093/eurheartj/ehaf193 40878294

[2] Regitz-Zagrosek V, et al. 2018 ESC Guidelines for the management of cardiovascular diseases during pregnancy. Eur Heart J. 2018;39(34):3165–241. 10.1093/eurheartj/ehy340 30165544

[3] van den Bersselaar LM, et al. Pregnancy and Delivery Outcomes in Vascular Ehlers-Danlos Syndrome: A Retrospective Multicentre Cohort Study. Bjog. 2026;133(3):463–70. 10.1111/1471-0528.18142 PMC12770062 40104886

[4] Sanghavi M, Rutherford JD. Cardiovascular physiology of pregnancy. Circulation. 2014;130(12):1003–8. 10.1161/CIRCULATIONAHA.114.009029 25223771

[5] Ramlakhan KP, et al. Pregnancy and cardiovascular disease. Nat Rev Cardiol. 2020;17(11):718–31. 10.1038/s41569-020-0390-z 32518358

[6] Estensen ME, Beitnes JO, Grindheim G, et al. Altered maternal left ventricular contractility and function during normal pregnancy. Ultrasound Obstet Gynecol. 2013;41(6):659–66. 10.1002/uog.12296 23001841

[7] Dhalwani NN, et al. Occurrence of fertility problems presenting to primary care. Hum Reprod. 2013;28(4):960–8. 10.1093/humrep/des451 23315065

[8] Rijs K, et al. How to set up a pregnancy heart team. Int J Cardiol Congenit Heart Dis. 2025;22:100635. 10.1016/j.ijcchd.2025.100635 PMC12664424 41322046

[9] Cornette J, van der Stok CJ, Reiss IKM, et al. Perinatal mortality and neonatal and maternal outcome per gestational week in term pregnancies. Acta Obstet Gynecol Scand. 2023;102(1):82–91. 10.1111/aogs.14467 PMC9780726 36263854

[10] Crawford K, Carlo WA, Odibo A, et al. Perinatal mortality and other severe adverse outcomes following planned birth at 39 weeks versus expectant management in low-risk women. EClinicalMedicine. 2025;80:103076. 10.1016/j.eclinm.2025.103076 PMC11803212 39927106

[11] Grobman WA, Rice MM, Reddy UM, T, et al. Labor Induction versus Expectant Management in Low-Risk Nulliparous Women. N Engl J Med. 2018;379(6):513–23. 10.1056/NEJMoa1800566 PMC6186292 30089070

[12] Hong J, Atkinson J, Roddy Mitchell A, et al. Comparison of Maternal Labor-Related Complications and Neonatal Outcomes Following Elective Induction of Labor at 39 Weeks of Gestation vs Expectant Management. JAMA Netw Open. 2023;6(5)e2313162. 10.1001/jamanetworkopen.2023.13162 PMC10182428 37171818

[13] Eke AC. An update on the physiologic changes during pregnancy and their impact on drug pharmacokinetics and pharmacogenomics. J. Basic and Clinical Phys Pharmacol. 2022;33(5):581–98. 10.1515/jbcpp-2021-0312 PMC9174343 34881531

[14] van Hagen IM, Roos-Hesselink JW, Ruys TPE, et M, et al. Pregnancy in Women With a Mechanical Heart Valve. Circulation. 2015;132(2):132–42. 10.1161/CIRCULATIONAHA.115.015242 26100109

[15] van der Zande JA, Ramlakhan KP, Sliwa K, et al. Pregnancy with a prosthetic heart valve, thrombosis, and bleeding. Eur Heart J. 2026;47(11):1318–35. 10.1093/eurheartj/ehaf265 PMC13016632 40237423

[16] Peters PNJ, van der Zande JA, De Backer J, et al. Pregnancy outcomes in women with heritable thoracic aortic disease. Eur Heart J. - Qual Care Clin Outcome. 2025;11(7):1091–107. 10.1093/ehjqcco/qcaf038 PMC12587288 40576452

[17] Murray ML, Pepin M, Peterson S, et al. Genet Med. 2014;16:874-80 10.1038/gim.2014.53 24922461

[18] Roos-Hesselink J, Baris L, Johnson M, et al. Pregnancy outcomes in women with cardiovascular disease. Eur Heart J. 2019;40(47):3848–55. 10.1093/eurheartj/ehz136 30907409

[19] Tita AT, Szychowski JM, Boggess K, et al. Treatment for Mild Chronic Hypertension during Pregnancy. N Engl J Med. 2022;386(19):1781–92. 10.1056/NEJMoa2201295 PMC9575330 35363951

[20] van Hagen IM, Thorne SA, Taha N, et al. Pregnancy Outcomes in Women With Rheumatic Mitral Valve Disease. Circulation. 2018;137(8):806–16. 10.1161/CIRCULATIONAHA.117.032561 29459466

[21] Baris L, Hakeem A, Moe T, et al. Acute Coronary Syndrome and Ischemic Heart Disease in Pregnancy. J Am Heart Assoc. 2020;9(15)e015490. 10.1161/JAHA.119.015490 PMC7792249 32750301

[22] Campens L, Baris L, Scott NS, et al. Pregnancy outcome in thoracic aortic disease data from the Registry Of Pregnancy And Cardiac disease. Heart. 2021;107(21):1704–9. 10.1136/heartjnl-2020-318183 PMC8522458 33468574

[23] Ramlakhan KP, Malhamé I, Marelli A, et al. Hypertensive disorders of pregnant women with heart disease. Eur Heart J. 2022;43(38):3749–61. 10.1093/eurheartj/ehac308 PMC9840477 35727736

[24] Ramlakhan KP, Roos-Hesselink JW, Basso T, et al. Perinatal outcomes after in-utero exposure to beta-blockers in women with heart disease. Int J Cardiol. 2024;410:132234. 10.1016/j.ijcard.2024.132234 38844094

[25] Tutarel O, Baris L, Budts W, et al. Pregnancy outcomes in women with a systemic right ventricle and transposition of the great arteries results from the ESC-EORP Registry of Pregnancy and Cardiac disease (ROPAC). Heart. 2022;108(2):117–23. 10.1136/heartjnl-2020-318685 PMC8717477 33911009

[26] Wander G, van der Zande JA, Patel RR, et al. Pregnancy in women with congenital heart disease: a focus on management and preventing the risk of complications. Expert Rev Cardiovasc Ther. 2023;21(8):587–99. 10.1080/14779072.2023.2237886 37470417

[27] Tutarel O, et al. Pregnancy Outcomes in Women After Arterial Switch Operation for Transposition of the Great Arteries. J Am Heart Assoc. 2021;10(1)e018176. 10.1161/JAHA.120.018176 PMC7955508 33350866

[28] van der Zande JA, Tutarel O, Ramlakhan KP, et al. Pregnancy outcomes in women with Ebstein’s anomaly. Open Heart. 2023;10(2). 10.1136/openhrt-2023-002406 PMC10407418 37550057

[29] Ruys TP, Roos-Hesselink JW, Hall R, et al. Heart failure in pregnant women with cardiac disease. Heart. 2014;100(3):231–8. 10.1136/heartjnl-2013-304888 24293523

[30] Botha TC, Pilcher GJ, Wolmarans K, et al. Statins and other lipid-lowering therapy and pregnancy outcomes in homozygous familial hypercholesterolaemia. Atherosclerosis. 2018;277:502–7. 10.1016/j.atherosclerosis.2018.05.038 30270091

[31] Chang JC, Chen YJ, Chen IC, et al. Perinatal Outcomes After Statin Exposure During Pregnancy. JAMA Netw Open. 2021;4(12)e2141321. 10.1001/jamanetworkopen.2021.41321 PMC8719244 34967881

[32] Karadas B, et al. Pregnancy outcomes following maternal exposure to statins. Br J Clin Pharmacol. 2022;88(9):3962–76. 10.1111/bcp.15423 35639354

[33] Majmundar M, Doshi R, Patel KN, et al. Prevalence, trends, and outcomes of cardiovascular diseases in pregnant patients in the USA: 2010-19. Eur Heart J. 2023;44(9):726–37. 10.1093/eurheartj/ehac669 36342471

[34] Lau ES, et al. Contemporary Burden of Cardiovascular Disease in Pregnancy. Circulation. 2025;152(15):1044–55. 10.1161/CIRCULATIONAHA.125.074692 PMC12614638 41048030

[35] De Backer J, Haugaa KH, Hasselberg NE, et al. 2025 ESC Guidelines for the management of cardiovascular disease and pregnancy. Eur Heart J. 2025;46(43):4462–568. 10.1093/eurheartj/ehaf193 40878294

